# Bell’s palsy at high altitude -- an unsuspected finding

**DOI:** 10.1186/s40779-016-0073-6

**Published:** 2016-05-12

**Authors:** K. V. S. Hari Kumar, K. P. Shijith, F. M. H. Ahmad

**Affiliations:** Departments of Endocrinology, Command Hospital, Chandimandir, 134107 India; Departments of Radiology, Command Hospital, Chandimandir, 134107 India; Departments of Neurology, Command Hospital, Chandimandir, 134107 India

**Keywords:** Bell’s palsy, Hyperparathyroidism, Vitamin D deficiency, Intracranial calcification

## Abstract

**Background:**

Bell’s palsy is a common condition seen in clinical practice. The aetiology of this condition is not clearly defined and neuroimaging is essential to exclude intracranial causes of infra-nuclear facial palsy.

**Case presentation:**

We report a young soldier, who presented with Bell’s palsy and neuroimaging revealed an unsuspected finding of multiple intracranial calcifications. Detailed evaluation revealed the additional diagnosis of vitamin D deficiency and secondary hyperparathyroidism due to lack of sun exposure at high altitude area.

**Conclusion:**

The health care practitioners, looking after the soldiers at high altitude areas should be aware of the measures to prevent vitamin D deficiency. Intracranial calcifications are uncommon in hyperparathyroidism and Bell’s palsy.

## Background

Soldiers working at high altitude areas are prone for unique disorders like acute mountain sickness, pulmonary edema and cerebral edema. These soldiers are often not exposed to the sun light and have to apply a sunscreen to prevent the ultraviolet ray induced damage to the skin. The supply chains are not very robust leading to the dependence on packaged food for many weeks to months. The packaged foods available in our country are not fortified with vitamins and minerals. We report a young soldier who presented with a seemingly trivial problem of Bell’s palsy, but detailed evaluation revealed the presence of an additional unsuspected disorder.

## Case presentation

**Fig. 1:**
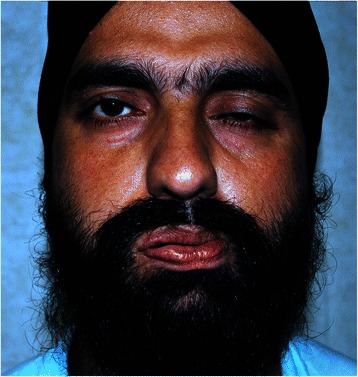
Clinical photograph showing the Bell’s palsy (Right)

**Fig. 2:**
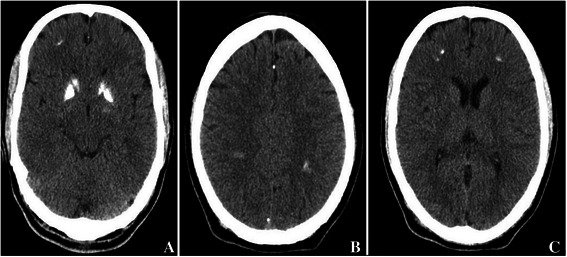
CT scan brain showing extensive intracranial calcifications involving basal ganglia (**a**), occipito-temporal (**b**) and fronto-parietal lobes (**c**)

A 31 year old soldier presented with a one week history of inability to close right eye, deviation of the face and pooling of saliva in the oral cavity. The patient denied history of fever, ear discharge and deafness. The patient was diagnosed as a case of Bell’s palsy and treated with oral acyclovir along with Prednisolone. A non-contrast CT scan brain showed the presence of multiple bilateral extensive intracranial calcifications especially involving the basal ganglia (Fig. 1). Hence, he was referred to us for detailed evaluation. At our centre, a history of leg pains and cramps on prolonged standing for over year duration was elicited. He denied past history of headache, seizures, neck surgery, psychiatric ailment or renal stone disease. None of the family members had a similar illness or ectopic calcification. He was working at an altitude of 15,000 feet with no sun exposure for over six months. Physical examination was normal and neurological examination showed lower motor neuron facial palsy of right side (Fig. 2). Clinical examination was unremarkable and Trousseau’s sign and Chvostek’s sign were negative. Other systemic examination was essentially normal.

His hematological and routine biochemical investigations were normal. His serum calcium (8.1 mg/dl), phosphorus (4.1 mg/dl) was normal with elevated alkaline phosphatase (185 U/L). His 25 hydroxy vitamin D (25OHD - 3.2 ng/dl) and intact parathyroid hormone (iPTH - 92 pg/ml) suggest the presence of secondary hyperparathyroidism (SHPT) due to vitamin D deficiency (VDD). Skeletal survey and ultrasonography abdomen were normal. He was diagnosed as a case of Bell’s palsy coexisting with SHPT and was treated with weekly vitamin D sachet (60,000 U) along with daily calcium (1,000 mg) supplements. Repeat investigations after 3 months revealed normal 25-OHD and iPTH with mild residual weakness of the facial muscles.

## Discussion

Bell’s palsy is an acute, unilateral, infranuclear facial palsy with no specific sex predilection. The disease has been reported in middle aged individuals residing at plains rather than at high altitude location. The disease is of unclear etiology and reactivation of the herpes simplex virus has been implicated by many researchers [1]. There is inflammation of the nerve leading to edema and focal ischemia of the facial nerve. The risk factors include obesity, diabetes, pregnancy and hypertension. The risk factors in our patient could be hypoxia induced neural edema coupled with vitamin D deficiency. Intracranial calcification also could have contributed for the same, but the neuroimaging did not reveal any evidence of calcification near the intracranial facial nerve.

Intracranial calcifications are seen in hypoparathyroidism, pseudohypoparathyroidism, hyperparathyroidism (especially due to renal failure), congenital infections (Toxoplasmosis), toxins (Carbon monoxide, lead), neoplasms (oligodendroglioma, craniopharyngioma, germ cell neoplasm), syndromes (Fahr’s, Cockyane) and mitochondrial cytopathies [2]. Intracranial calcifications are seen physiologically also, in certain areas of brain like pineal gland, choroid plexus, falx, tentorium and sagittal sinus. Congenital calcifications are seen in Sturge-Weber syndrome, Tuberous sclerosis, neurofibromatosis, Cockayne and Gorlin syndromes.

SHPT leading to metastatic calcification is reported commonly in patients with chronic kidney disease [3]. SHPT due to vitamin D deficiency leading to extensive intracranial calcification has been rarely reported [4]. The unique features of our patient include a presentation with Bell’s palsy and SHPT due to VDD. Similar to the other published report, our patients also had very low levels of 25hydroxy vitamin D and evidence of SHPT. The pathogenesis behind the ectopic calcification is not very clear. The widely accepted theories include elevated calcium-phosphorus product, increased tissue sensitivity and individual susceptibility [5]. The tissues express the osteoblastic phenotype and the ectopic calcification is an actively determined process rather than the passive mineral deposition. Metastatic calcification is seen in various internal organs like heart, lungs, kidney, vasculature and external genitalia including penis and scrotum. The presence of vascular calcification leading to ischemic necrosis and cutaneous gangrene is known as the calciphylaxis, which is an indicator of high mortality [6].

Vitamin D plays an important role in skeletal and extra skeletal health. The presence of VDD is associated with the massive calcifications of the basal ganglia, cortex and cerebellum. Reports from the mice showed presence of numerous calcium enriched laminated bodies in the basal ganglia in vitamin D receptor knockout mice [7]. The treatment is often unsatisfactory due to the unclear pathogenesis of the condition. The management options include calcitriol and phosphate binders like sevelamer.

## Conclusion

In conclusion, we report a case of Bell’s palsy with secondary hyperparathyroidism. The soldiers working at high altitude areas should be educated about the requirement of adequate vitamin D intake. The packaged foods should be fortified with vitamin D to improve the skeletal health of these soldiers.

## Consent

“Written informed consent was obtained from the patient for publication of this Case Report and any accompanying images. A copy of the written consent is available for review by the Editor-in-Chief of this journal.”
